# The Effect of Servant Leadership on Work Resilience: Evidence from the Hospitality Industry during the COVID-19 Period

**DOI:** 10.3390/ijerph20021322

**Published:** 2023-01-11

**Authors:** Zhenyao Cai, Yimin Mao, Ting Gong, Ying Xin, Jiayun Lou

**Affiliations:** SILC Business School, Shanghai University, 20 Chengzhong Road, Shanghai 201800, China

**Keywords:** servant leadership, emotional exhaustion, state resilience, work complexity, conservation of resources theory

## Abstract

The COVID-19 pandemic is a tremendous crisis for public health, which also has a profound impact on business and social activities because many countries restrict travel and social gatherings to avoid the spread of COVID-19. Workers suffer from mental health problems including depression and anxiety due to the uncertain work environment. Hence, psychological resilience, a positive psychological response to these challenges, is essential to the success of employees and companies. Drawing on the conservation of resources theory (COR), this paper investigates how the leadership style (i.e., servant leadership) enhances the work resilience of hospitality employees through two time-lagged empirical studies. Specifically, study 1 demonstrates a positive relationship between servant leadership and employees’ work resilience. Study 2 replicates study 1’s result and further demonstrates that emotional exhaustion mediates the relationship between servant leadership and employees’ work resilience. Furthermore, study 2 finds a significant moderating effect of job complexity. The findings of this paper provide empirical evidence for practitioners to manage employees’ resilience and psychological resources.

## 1. Introduction

The pandemic of COVID-19 is a tremendous crisis for public health. According to WHO [1], over 600 million people were infected. Because many countries restrict travel and social gatherings to avoid the spread of COVID-19, many citizens have suffered significant mental health problems, including depression and anxiety [2]. Researchers show that 27.5% of the respondents suffered from anxiety disorders, 29.3% suffered from depression, and 30.0% suffered from sleep disorders during the pandemic [3].

The pandemic also has a profound impact on business industries. One of the worst affected industries is the hospitality industry, due to the lockdown restrictions. Hospitality employees are in fear of being infected and fired when they are in this uncertain work environment [4,5,6]. Thus, many hospitality workers and students decided to change their job and are unwilling to develop their careers in the hospitality industry [7,8]. Hence, understanding how hospitality workers effectively face crises and grow work resilience is essential to researchers and practitioners in the hospitality field [9,10,11]. In the workplace, leaders play important roles in engaging job behaviors and reducing the anxiety of hospitality employees during the pandemic [12,13,14,15]. The negative impact of the pandemic motivates researchers to consider what leaders can do to help hospitality employees build resilience to the crisis.

Building on the conservation of resource theory, this paper investigates the effect of servant leadership on hospitality employees’ work resilience, as well as the mediating role of emotional exhaustion and the moderating role of job complexity. To test the hypotheses, this paper conducts two separate studies using a time-lagged data collection procedure. Specifically, study 1 demonstrates a positive relationship between servant leadership and employees’ work resilience. Study 2 replicates study 1’s result and further demonstrates that emotional exhaustion mediates the relationship between servant leadership and employees’ work resilience. It also finds a significant moderating effect of job complexity. This research design provides two strengths of the paper. On the one hand, the effect of servant leadership on work resilience was supported by two separate data, providing robust evidence in the hospitality industry. On the other hand, the time-lagged data collection procedure helps rule out the potential threats of common method variance considering that all the studied variables were surveyed and evaluated from the employees’ perspectives [16]. 

This paper intends to contribute to the hospitality literature from three perspectives. First, it enriches the empirical research on the antecedent of work resilience in the hospitality area. Previous research in the hospitality literature either focuses on organizational-level resilience or the moderating role of employees’ resilience [17,18,19,20]. Very few articles investigate the antecedents of employees’ resilience during the COVID-19 period. Because resilience is a key predictor of employees’ stress, job satisfaction, and turnover intention, researchers call for more empirical studies on the antecedents of work resilience of hospitality employees during and after the pandemic [21]. Second, it tests how servant leadership enhances work resilience, contributing to the understanding of the underlying mechanism between leadership and work resilience. Previous research mainly focuses on social exchange, social learning, and social identity theories to explain how servant leadership affects followers’ behaviors [22,23,24]. In a recent review of the servant leadership literature, the authors call for more research to study the mechanism of the conservation of resources theory [25]. Therefore, this paper aims to respond to their call and empirically test the mechanism between servant leadership and employees’ work resilience from the COR perspective. Third, previous studies on the moderating effects of how servant leadership influences followers’ behaviors primarily focus on the leader’s and followers’ personal characteristics and motivation [26,27]. It is essential to understand how the job design factor moderates the effect of servant leadership because job design factors are important sources of job resources in the workplace. This paper extends the boundary conditions of how the job design factor (i.e., job complexity) strengthens the effect of leadership on work resilience. It responds to the call for more research on how job complexity moderates the relationship between servant leadership and follower outcomes [25]. 

## 2. Literature Review and Hypotheses Development

### 2.1. The Effect of Servant Leadership on Work Resilience

Work resilience refers to “the capacity to manage the everyday stress of work and remain healthy, rebound and learn from unexpected setbacks and prepare for future challenges proactively” [28]. It is a positive psychological resource supporting employees during the pandemic. A high work resilience normally leads to positive psychological outcomes, such as job satisfaction, psychological well-being, and work engagement [29,30]. Researchers have demonstrated that different leadership styles play vital roles in predicting employees’ work resilience [31,32,33]. 

Servant leadership is defined as leader behaviors that prioritize the needs of employees and other related stakeholders in the community [34,35]. Grounded in other orientations and serving the community, it attracts great attention in the hospitality literature (see the review in Bavik [36]). Servant leadership is conceptually distinguished from other leadership styles in the broader leadership literature [37,38]. For example, although transformational leadership also focuses on followers’ needs, servant leadership is conceptually different from transformational leadership [25,39]. Researchers indicate that servant leadership focuses on the psychological needs of followers as a goal in itself, whereas transformational leadership focuses on these needs for the purpose of achieving organizational goals [40]. Similar to authentic leadership, servant leaders are also authentic and true to their followers. These two constructs are conceptually different because servant leaders are driven by the sense of calling and prosocial motivation, not for the sake of being authentic [25,41]. Researchers indicate that some behavioral perspectives are similar to ethical leadership. Servant leadership is different from ethical leadership because ethical leadership provides little attention to the development of the followers [25,42]. Given the above characteristics of servant leadership, it is a potential antecedent of employees’ work resilience because it focuses more on the followers’ needs for the sake of the followers. 

Researchers indicate that a positive work environment is an important source of work resilience [43]. During the pandemic, the characteristics of servant leaders, such as humility, interpersonal acceptance, and empowering and developing people, provide various supports to the employees [39]. Servant leaders can create a positive working climate and stimulate positive work emotions and psychological resources. In support of this argument, previous research has found that servant leaders can promote a service climate [44]. When employees internalize psychological resources, they are more likely to gain strong work resilience and respond to challenges effectively. Researchers have found that servant leadership is a key predictor of job resources [45].

**Hypothesis 1.**  *Servant leadership has a positive relationship with employees’ work resilience.*

### 2.2. The Effect of Servant Leadership on Emotional Exhaustion

According to the conservation of resources theory, individuals tend to acquire, preserve and maintain valuable resources, which are vital in predicting work outcomes [46,47]. Positive work events can stimulate work-related resources, and negative work events deplete employees’ resources [46]. These resources are essential for the employees to override aversive emotional states [48]. Emotional exhaustion, a dimension of burnout, refers to a psychological state of emotional depletion in the workplace [49]. It is likely to occur when employees expend substantial resources to handle negative situations [50].

During the pandemic, leaders can help minimize employees’ potential anxiety by providing them with a supportive and safe working environment [51]. Researchers have indicated that employees see servant leadership as a positive resource [52]. The value of “employee first” by servant leaders can enhance employees’ psychological safety and positive emotions. Positive emotions can further reduce stress and maintain psychological resources. In this case, employees are less likely to perceive emotional exhaustion. Previous research has demonstrated the role of servant leadership in reducing employees’ emotional exhaustion [53].

**Hypothesis 2.**  *Servant leadership has a negative relationship with employees’ emotional exhaustion.*

### 2.3. The Effect of Emotional Exhaustion on Work Resilience

Previous researchers primarily focused on the effect of work resilience on emotional exhaustion because they investigated the trait perspective of work resilience. Trait resilience is an individual characteristic that enhances one’s ability to respond to negative experiences and events [54]. Unlike previous research, this paper focuses on the state perspective of work resilience. According to the conservation of resources theory, depleted employees tend to protect their limited resources and prevent further depletion of the remaining resources [55]. Because psychological resources are important sources of state work resilience [56], once psychological resources are depleted excessively, work resilience will be reduced. Previous research has found that when employees detach from work, they are more likely to maintain a high level of state resilience [57]. Therefore, this paper argues that when hospitality employees perceive emotional exhaustion, they tend to preserve their limited resources, leading to a lower level of work resilience.

**Hypothesis 3.**  *Emotional exhaustion has a negative relationship with employees’ work resilience.*

### 2.4. Mediating Effect of Emotional Exhaustion

We have hypothesized the direct relationship between servant leadership and work resilience (Hypothesis 1), the relationship between servant leadership and emotional exhaustion (Hypothesis 2), and the relationship between emotional exhaustion and work resilience (Hypothesis 3). Taken together, we further hypothesize a mediating effect between servant leadership and work resilience through emotional exhaustion. We argue that when servant leaders provide support for hospitality employees, employees are more resilient to the work because they can gain external resources and are less likely to perceive emotional exhaustion.

**Hypothesis 4.**  *Emotional exhaustion mediates the relationship between servant leadership and work resilience.*

### 2.5. The Moderating Effect of Job Complexity

The core definition of job complexity refers to jobs that are mentally challenging and require the employees to apply complex skills in the work [58,59]. Complex jobs are also described by ambiguity and a lack of structure [60]. On the other hand, simple jobs are usually well-defined and routine [60]. Job complexity has been found to be relevant to emotional exhaustion [61]. We argue that employees who work in less complex jobs are more likely to have lower work resilience when they are emotionally exhausted.

Complex jobs can foster employees’ capacity to master jobs because employees need to make additional efforts to meet the challenges and difficulties [62,63]. Therefore, job complexity is seen as an important situational resource for employees [64], which can mitigate the negative effect of emotional exhaustion. In this case, job complexity can enhance employees’ capacity to protect their personal resources [61] and counteract the resource loss associated with emotional exhaustion at the workplace. Previous research finds that employees whose work offers job complexity suffer less from emotional exhaustion [61].

**Hypothesis 5.**  *Job complexity moderates the relationship between emotional exhaustion and work resilience. When job complexity is low, the relationship between emotional exhaustion and work resilience is significant.*

### 2.6. Moderated Mediation

Taking the above hypotheses together, we further hypothesize a conditional indirect effect between servant leadership and employees’ work resilience through emotional exhaustion.

**Hypothesis 6.**  *Job complexity moderates the indirect effect between servant leadership and work resilience through emotional exhaustion. When job complexity is high, the indirect effect is not significant.*

**Figure 1:**
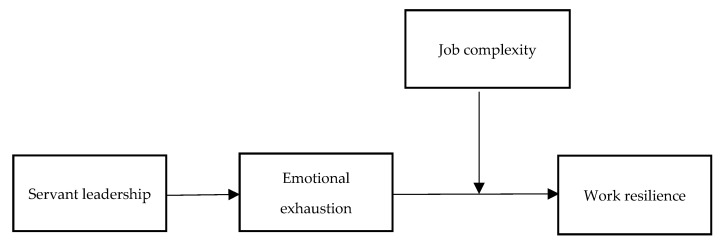
Theoretical model.

The research model of the study is shown in Figure 1.

## 3. Method

To test the hypotheses, we conducted two separate studies. Specifically, study 1 collected time-lagged data to test the main effect of servant leadership on work resilience (Hypothesis 1). Study 2 collected another set of data to replicate the findings of study 1 and further test the mediating and moderating effects and the moderated mediation effect (Hypotheses 2–6).

### 3.1. Study 1

#### 3.1.1. Participants and Procedure

In Study 1, we collected data from frontline hospitality employees working in two large hotels in China using the convenience sampling method. The data collection procedure was supported by two Chinese hotels. With the help of human resource managers, we created a pool of potential respondents including all the full-time frontline employees. Due to the pandemic, we used the online survey platform to distribute the questionnaires to the potential respondents. To avoid common method bias [16], we applied the time-lagged design and collected data in two waves, three weeks apart. Specifically, we asked for servant leadership in the first wave and work resilience in the second wave. Before the data collection, the potential respondents were informed that this study was only used for academic purposes and would keep their personal information confidential. A total of 200 questionnaires were received in the first wave. In wave two, the second set of questionnaires was distributed to the respondents who participated in the first wave. Finally, 177 valid matching questionnaires were returned, with a response rate of 88.5%. Of the samples, 18.7% were male employees. Furthermore, 24.9% were aged 25 and below, 49.7% were aged 26–30, and 25.4% were aged 31 and above. In total, 40.7% had a university degree or above.

#### 3.1.2. Measures

The questionnaires were in Chinese. We adopted the back-translation procedure to ensure the accuracy of the translation. All variables were rated using a 5-point Likert-type scale ranging from 1 (strongly disagree) to 5 (strongly agree).

Servant leadership was measured by the 7-item scale of Liden et al. [65]. Sample items included “My leader puts my best interests ahead of his/her own” and “My leader gives me the freedom to handle difficult situations in the way that I feel is best “ (Cronbach’s alpha = 0.74).

Work resilience was adapted from the 6-item scale of Smith et al. [66]. Sample items included “In recent days, while at work, it does not take me long to recover from a stressful event” and “In recent days, while at work, I usually come through difficult times with little trouble” (Cronbach’s alpha = 0.86).

Moreover, we also controlled employees’ gender, age, and educational level in the regression analysis.

#### 3.1.3. Results

Prior to testing the hypotheses, we conducted a confirmatory factor analysis to evaluate the convergent and discriminant validity. To maintain an acceptable item-to-sample-size ratio, we adopted the parceling strategy by creating three parcels for servant leadership and work resilience, respectively. Finally, the two-factor model had an acceptable fit (Chi-square = 28.34, *df* = 8, RMSEA = 0.12, CFI = 0.95, TLI = 0.90) and all the factor loadings were significant, demonstrating the convergent validity. Moreover, the discriminant validity was assessed using Fornel and Larcker’s procedure [67]. The square root of average variances extracted (i.e., 0.69 for servant leadership and 0.82 for work resilience) were larger than the correlations between servant leadership and work resilience, demonstrating good discriminant validity.

Table 1 shows the means, standard deviations, and correlations for all the variables. The results showed that there is a significant positive correlation between servant leadership and working state resilience (r = 0.40, *p* < 0.01), and there is a significant correlation between the two variables, which provides preliminary support for regression analysis.

To test Hypothesis 1, we regressed servant leadership on work resilience and the control variables. The results shown in Table 2 indicate that servant leadership has a positive and significant relationship with work resilience (β = 0.40, *p* < 0.001). Hypothesis 1 was supported. 

### 3.2. Study 2

#### 3.2.1. Participants and Procedure

In Study 2, data were collected through WJX, an online participant recruitment and data collection platform in China. It is popular to use a third-party recruitment platform to collect survey data [68,69]. Employees working in the catering, hotel, and tourism industries were recruited to participate in the study. To avoid common method variance, the data collection procedure was separated into two waves, three weeks apart. Specifically, servant leadership and emotional exhaustion were collected in the first wave, and job complexity and work resilience were collected in the second wave. A total of 632 respondents returned the questionnaires in the first wave. Three weeks later, the second set of questionnaires was sent to the respondents who participated in the first wave. Finally, 349 valid questionnaires were included for further analysis, with a response rate of 55.2%. Of the samples, 52.4% were female. 16.9% were aged 25 and below, 67.3% were aged 26–35, and 15.8% were aged 36 and above. Furthermore, 86.2% had a university degree.

#### 3.2.2. Measures

Similar to study 1, we adopted the back-translation procedure to ensure the accuracy of the translation. All variables were rated using a 5-point Likert-type scale ranging from 1 (strongly disagree) to 5 (strongly agree).

Servant leadership was measured by Ehrhart’s 14-item scale [70]. Sample items include Representative questions such as: “My leader makes the personal development of department employees a priority” and “My leader works hard at finding ways to help others be the best they can be” (Cronbach’s alpha = 0.80).

Emotional exhaustion was measured by Maslach and Jackson’s 9-item scale [49]. Sample items included representative items such as: “I feel used up at the end of the workday” and “I feel burned out from my work” (Cronbach’s alpha = 0.92).

Job complexity was measured by the 3-item scale of Cammann et al. [71]. Representative questions included “My job is very complex” and “My job is such that it takes a long time to learn the skills required to do the job well” (Cronbach’s alpha = 0.70).

Work resilience was measured by the 6-item scale of Smith et al. [66]. Sample items included “In recent days, while at work, I tend to bounce back quickly after hard times” and “In recent days, while at work, I usually come through difficult times with little trouble” (Cronbach’s alpha = 0.76).

Furthermore, similarly to study 1, we also included employees’ age, gender, and educational level as control variables.

#### 3.2.3. Results

First, we conducted a confirmatory factor analysis to evaluate the convergent and discriminant validity. To maintain the item-to-sample-size ratio, we created five parcels for servant leadership, three parcels for emotional exhaustion, and three parcels for job complexity. The five-factor model had an acceptable fit (Chi-square = 255.11, *df* = 160, RMSEA = 0.04, CFI = 0.96, TLI = 0.96) and all the factor loadings were significant, demonstrating the convergent validity. Moreover, the square root of average variances extracted, ranging from 0.59 to 0.88, were all larger than the correlation between the corresponding variable and any other variables. Thus, the discriminant validity was confirmed, and all the variables were included for further analysis.

Table 3 shows the means, standard deviations, and correlations. Servant leadership was negatively correlated to emotional exhaustion (r = −0.44, *p* < 0.01), emotional exhaustion was negatively correlated to work resilience (r = −0.25, *p* < 0.01), and servant leadership was positively correlated to work resilience (r = 0.35, *p* < 0.01).

Hierarchical linear regression was used to test Hypotheses 1, 2, and 3. As shown in Table 4, servant leadership has a positive relationship with work resilience (β = 0.34, *p* < 0.001) and a negative relationship with emotional exhaustion (β = −0.44, *p* < 0.001). Moreover, emotional exhaustion has a negative relationship with work resilience (β = −0.24, *p* < 0.01). Hence, Hypotheses 1, 2, and 3 were supported.

To test Hypothesis 4, the mediating effect of emotional exhaustion between servant leadership and work resilience, we adopted PROCESS macro 3.5 [72]. Specifically, bootstrapping analysis using Model 4 of PROCESS macro was performed with 5000 re-samples at the 95% bias-corrected confidence interval. As shown in Table 5, the indirect effect between servant leadership and work resilience through emotional exhaustion was significant (effect = 0.06, SE = 0.03, bias-corrected confidence interval = 0.001, 0.13), supporting Hypothesis 4.

**Figure 2:**
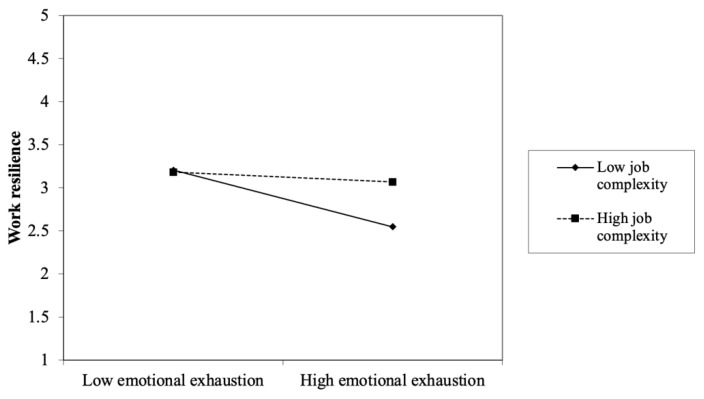
The moderating effect of job complexity.

Hypothesis 5 examines the moderating role of job complexity on the relationship between emotional exhaustion and work resilience. To test the moderating effect, we followed the procedure by Aiken and West [73] to create the interaction term via the production of mean-centered emotional exhaustion and work resilience. As shown in Table 6, the interaction term was positively related to work resilience (β = 0.14, *p* < 0.01). The result of the simple slope test (see Figure 2) indicated that the relationship between emotional exhaustion and work resilience was significant when job complexity was low (β = −0.29, *p* < 0.01) but not significant when job complexity was high (β = −0.09, *p* > 0.05). Thus, Hypothesis 5 was supported.

To test Hypothesis 6, we conducted bootstrapping analysis with 5000 re-samples via Model 14 of PROCESS macro 3.5. As shown in Table 5, the indirect effect between servant leadership and work resilience through emotional exhaustion was significant when job complexity was low at the 95% bias-corrected confidence interval (effect = 0.15, SE = 0.04, bias-corrected confidence interval = 0.07, 0.24), but not significant when job complexity was high (effect = −0.01, SE = 0.04, bias-corrected confidence interval = −0.09, 0.08). The index of moderated mediation was significant (effect = −0.10, SE = 0.03, bias-corrected confidence interval = −0.17, −0.03). Therefore, Hypothesis 6 was supported.

## 4. Discussion

The results supported all the hypotheses of the research model. Specifically, study 1 demonstrated that servant leadership has a positive relationship with employees’ work resilience. Study 2 replicated the results of study 1 and further demonstrated the mediating effect of emotional exhaustion on the relationship between servant leadership and work resilience. Moreover, study 2 supported the hypothesis that job complexity moderated the relationship between emotional exhaustion and work resilience and further moderated the mediating effect.

### 4.1. Theoretical and Practical Implications

This paper makes three important theoretical contributions to the literature on servant leadership and work resilience. First, the findings of this paper contribute to the literature by demonstrating that servant leadership can enhance employees’ work resilience. Leadership is an important antecedent of work resilience. For example, previous research has indicated that transformational and transactional leadership can enhance work resilience [74]. In addition, the findings also add to the knowledge of the individual outcomes of servant leadership. This paper extends the current literature by showing the effect of servant leadership on work resilience.

Second, this paper extends the understanding of the underlying mechanism of the relationship between servant leadership and work resilience. Specifically, we draw on the conservation of resources theory and investigate the mediating role of emotional exhaustion. To our knowledge, this is the first empirical study examining the mediating effect of the relationship between servant leadership and work resilience from the resource perspective. It extends to our knowledge of how the employees respond to the servant leaders and become more resilient to challenges in the workplace.

Third, this paper extends the research of the boundary conditions on the relationship between emotional exhaustion and work resilience. In addition, it also finds the moderated mediating effect between servant leadership and work resilience through emotional exhaustion. Integrating the job design literature, this paper demonstrated that the job design factors (i.e., job complexity) could weaken the negative effect of emotional exhaustion on work resilience. Furthermore, the significant moderated mediating effect implies that job complexity is an important boundary condition. 

Fourth, the findings of this paper provide implications for how leadership and organizational factors affect employees’ work resilience in the Chinese cultural context. Researchers have demonstrated the effectiveness of servant leadership in the Chinese culture [75,76,77]. The findings of this paper contribute to the literature by providing empirical evidence of how organizational efforts, such as promoting a servant leader and better job design, affect employees’ work resilience. 

In addition to the theoretical implications, the findings of this paper also have two implications for practitioners. First, the results imply the importance of servant leaders for employees in the hospitality industry. During the pandemic, employees in the hospitality industry have suffered great anxiety and pressure because of the uncertainty in their work environment. To enhance the work resilience of hospitality employees, companies can consider selecting and training more servant leaders. Second, the findings also provide implications for the organization to provide better support during the pandemic. For example, organizations may consider making policies to promote servant leaders and enhance the positive effect of servant leadership. Third, managers should pay more attention to the emotions of employees during the pandemic because when employees perceive high emotional exhaustion, they will be less resilient to work. It is more important in the hospitality industry when most of the jobs are routine and not complex, because employees who conduct routine jobs, rather than complex jobs, are more likely to be less resilient when they feel emotionally exhausted. 

### 4.2. Limitations and Future Research

This paper includes several limitations. First, to avoid common method variance, this paper collected time-lagged data in two studies. However, servant leadership and emotional exhaustion were still collected at the same time in study 2, making it difficult to demonstrate a clear causality between servant leadership and emotional exhaustion. Future research may consider collecting these variables at different times. Furthermore, future research may also consider collecting data from different sources. Second, this paper does not consider the work conditions of the respondents. During the pandemic, many hotels were used as quarantine hotels. Employees working in quarantine hotels may perceive more anxiety and pressure and are more likely to perceive emotional exhaustion. Future research may consider more control variables in the analysis. Third, this paper does not consider the size of the company because large companies normally have an established human resource system to support their employees. 

In addition to the limitation, future research may also extend the research model in four ways. First, future research may explore more underlying mechanisms. This paper tests the underlying mechanisms from the resource perspective. Other mechanisms may also explain why servant leadership can enhance work resilience. For example, according to social exchange theory [78], servant leadership may stimulate the perception of organizational support, leading to work resilience. Moreover, the social cognitive process may also be relevant because servant leaders providing support for the employees may enhance the self-efficacy of employees, leading to high work resilience. Third, future research can consider more boundary conditions of job design. For example, job interdependence may be relevant. When employees work together and interact more with their colleagues, they may be more able to adjust their emotions to anxiety and pressure. Fourth, although this paper demonstrates a significant relationship between servant leadership and work resilience, future research may consider whether the company can benefit from the enhancement of employees’ work resilience. Furthermore, because the data were collected from the hospitality industry, the findings are very context specific. Because the frontline employees working in the hospitality industry need to have frequent face-to-face interactions with many customers in their daily jobs, the mechanisms of these frontline employees may be different from the employees working in other industries. Hence, future research may also consider whether our conclusions are applicable to other industries. 

## 5. Conclusions

Employees working in the hospitality industry have suffered great anxiety and pressure during the COVID-19 pandemic. Understanding how to enhance hospitality employees’ work resilience is important to the hospitality field’s managers and researchers. Therefore, this paper collected two separate data and investigated the effect of servant leadership on work resilience, as well as the underlying mechanism of this effect. Moreover, the findings demonstrate that job complexity is an important boundary condition. The findings of this paper provide theoretical implications to the literature. It also provides evidence for practitioners to formulate better management policies for hospitality employees during the pandemic.

## Figures and Tables

**Table 1:** Means, standard deviations, and correlations.

Variable	M	SD	1	2	3	4
1. Gender	1.81	0.39				
2. Age	28.31	3.98	0.15			
3. Education level	4.35	0.59	0.11	−0.36 **		
4. Servant leadership	4.41	0.86	0.06	0.03	−0.25 **	
5. Work resilience	4.74	0.94	−0.12	0.08	−0.15 *	0.40 **

Notes: N = 177; * *p* < 0.05, ** *p* < 0.01.

**Table 2:** Linear regression results.

Variable	Standardized β	R^2^
1. Gender	−0.16	0.19
2. Age	0.09	
3. Education level	0.00	
4. Servant leadership	0.40 ***	

Notes: Dependent variable = working state resilience; N = 177; *** *p* < 0.001.

**Table 3:** Means, standard deviations, and correlations.

Variable	M	SD	1	2	3	4	5	6	7
1. Gender	1.52	0.50							
2. Age	3.01	0.64	−0.09						
3. Education level	3.01	0.37	−0.03	0.09					
4. Servant leadership	3.74	0.52	0.02	−0.02	0.10	(0.64)			
5. Emotional exhaustion	2.31	0.87	0.00	−0.10	−0.09	−0.44 **	(0.88)		
6. Job complexity	3.46	0.73	−0.05	0.02	0.04	0.16 **	0.02	(0.66)	
7. Work resilience	3.54	0.66	−0.04	0.10	0.09	0.35 **	−0.25 **	0.12 *	(0.59)

Notes: N = 349; * *p* < 0.05, ** *p* < 0.01. The square root of average variances extracted from the focal variables is presented in brackets.

**Table 4:** Hierarchical linear regression.

Variable	Work Resilience	Emotional Exhaustion	Work Resilience
1. Gender	−0.04	−0.01	−0.04
2. Age	0.01	−0.11 *	−0.02
3. Education level	0.06	−0.05	0.07
4. Servant leadership	0.34 ***	−0.44 ***	-
5. Emotional exhaustion	-	-	−0.24 **
R^2^	0.13	0.21	0.07

Notes: N = 349; * *p* < 0.05, ** *p* < 0.01, *** *p* < 0.001.

**Table 5:** Bootstrapping test results.

		Effect	SE	95% Confidence Interval	
				LLCI	ULCI
Mediator: emotional exhaustion	The mediating effect of emotional exhaustion	0.06	0.03	0.001	0.13
Moderator: job complexity	Low job complexity	0.15	0.04	0.07	0.24
	High job complexity	−0.01	0.04	−0.09	0.08
	Index of Moderated mediation	−0.10	0.03	−0.17	−0.03

**Table 6:** Hierarchical regression results.

Variable	Model 1	Model 2	Model 3
Gender	−0.05	−0.05	−0.03
Age	−0.02	−0.02	−0.02
Education level	0.12	0.11	0.08
Emotional exhaustion	−0.24 ***	−0.24 ***	−0.19 **
Work complexity		0.12 *	0.12 **
Emotional exhaustion × work complexity			0.14 **
R^2^	0.07	0.08	0.10
ΔR^2^	0.07 ***	0.01 *	0.02 **

Notes: Dependent variable = work resilience; N = 349; * *p* < 0.05, ** *p* < 0.01, *** *p* < 0.001.

## Data Availability

The data presented in this study are available upon request from the corresponding author.
